# Physiologically Based Structure of Mean Residence Time

**DOI:** 10.1100/2012/610631

**Published:** 2012-04-01

**Authors:** Mária Ďurišová

**Affiliations:** The Institute of Experimental Pharmacology and Toxicology, Slovak Academy of Sciences, 84104 Bratislava, Slovakia

## Abstract

A mean residence time (MRT) is an important pharmacokinetic parameter. To the author's knowledge, however, a physiologically based structure of MRT (thereafter MRT structure) has not been published so far. Primarily this is because MRT structures cannot be identified by traditional pharmacokinetic methods used for the determination of MRT. Therefore, tools from the theory of linear dynamic systems were used for the structural identification of MRT in this study. The MRT structure identified is physiologically meaningful. Accordingly, it seems that the MRT structure identified may contribute to already established knowledge about MRT.

## 1. Introduction

A mean residence time (MRT) is an important pharmacokinetic parameter. However, to the author's knowledge, a physiologically based structure of MRT (thereafter MRT structure) has not been published so far. Primarily this is because MRT structures cannot be identified by traditional pharmacokinetic methods, commonly used to determine MRT. Therefore, tools from the linear dynamic system theory were used for the structural identification of MRT in this study [1].

 Drug disposition is a composite procedure arising from the complexity and diversity of interactions between the drug administered, physiological mechanisms, and various exogenous factors. Furthermore, drug disposition is controlled to a greater extent by several dynamic pharmacokinetic processes [2]. For that reason, several studies described investigations of drug disposition with the aid of dynamic systems, using the following steps: (1) a construction of an ADME-related dynamic system, (2) a development of a mathematical model of the system constructed, for example, [3–7]. ADME-related dynamic systems are mathematical constructs (working tools) without any pharmacokinetic relevance. The meaning of the acronym ADME is explained in many pharmacokinetic studies, for example, [8]. In the present study, the ADME-related dynamic system was simply called the system.

 There are the following highly significant differences in terminology between pharmacokinetics and this study, which may trigger misunderstanding for readers: The difference between the physiological nature of information conveyed by a physiological system and the functional nature of information conveyed by the system used in this study. The difference in the use of the term “dynamic”. In pharmacokinetics, the term “dynamic” is commonly used in expressions describing drug actions. In this study, the term “dynamic” was used to indicate that the system or process changes over time.

## 2. Methods

The structural identification of MRT was performed employing these steps.

(1) The introduction of the following approximate assumptions: an instantaneous mixing of the drug with blood, a uniform drug distribution [9]. A linear drug disposition [9–11]. The liver is the only eliminating organ of significance for the drug administered [12].

(2) The use of the method described previously [4, 7] and a theoretical example in which it was assumed that the drug was administered in an intravenous bolus dose to a hypothetical subject.

(3) The formalization of drug disposition using the system *H*_*iv*_, created with the following configuration: the drug administration was considered as an input to the system *H*_*iv*_, and the concentration-time profile of the drug in arterial blood was considered as an output of the system *H*_*iv*_.

 (4) The development of a circulatory model of the system *H*_*iv*_, taking into account the fact that drug disposition can be regarded as a result of repetitive passes of the drug around the blood circulation, for example, [10, 13–16].

(5) The determination of the transfer function *H*_*iv*_(*s*) of the system *H*_*iv*_, for example, [3–7].

(6) The derivation of the general equation (1) for the determination of MRT:

(1) $$\text{MRT}=\frac{\lim_{s\to 0}\left(dH_{iv}\left(s\right)/ds\right)}{\lim_{s\to 0}H_{iv}\left(s\right)},$$

using the transfer function *H*_*iv*_(*s*) (*s* is the Laplace variable) and all assumptions made until now, for example, [3–7, 11].

(7) The use of the general equation (1), the circulatory model developed, and the method described previously [4] to identify the MRT structure.

## 3. Results and Discussion

**Figure 1:**
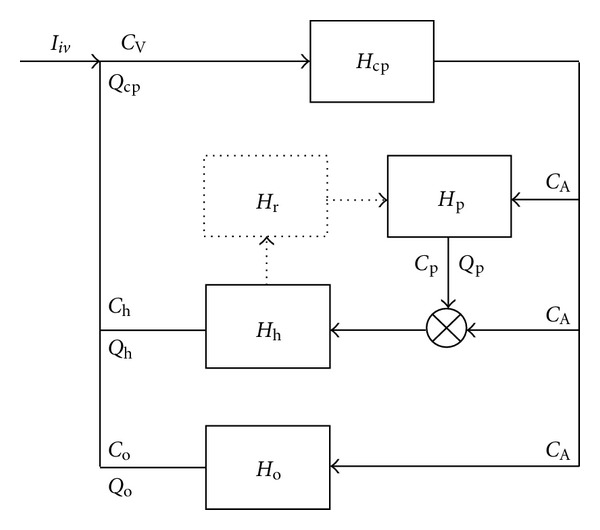
Circulatory model of the system *H*_*iv*_ describing drug disposition in a human body after an intravenous bolus dose of a drug. The drug administration is denoted by *I*_*iv*_. The concentration-time profile of the drug in arterial blood denoted with *C*_*A*_. The concentration-time profile of the drug in venous blood denoted with *C*_*V*_. The cardiopulmonary subsystem, describing the drug transport through the heart and lungs, is denoted by *H*_*cp*_.  *H*_*p*_ is the portal-venous subsystem describing the portal transport of the drug. *H*_*h*_ is the hepatic-portal subsystem describing the hepatic transport of the drug. *H*_*o*_ is the subsystem describing the drug transport through noneliminating tissues. *H*_*r*_ is the subsystem describing the enterohepatic cycling. The subsystem *H*_*r*_ is shown by a dotted line, to indicate that the enterohepatic cycling is not always present. The symbol ⊗ denotes a summation operator. *Q*_*cp*_, *Q*_*p*_, *Q*_*h*_, *Q*_*o*_ are blood flows in the subsystems specified by the subscripts.

The developed circulatory model of the system *H*_*iv*_ is depicted in Figure 1. As seen, major body organs are lumped into subsystems of the system *H*_*iv*_, that is, into somewhat independent parts of the system *H*_*iv*_, for example, [13–17]. The model takes into account the fact that drug disposition can be regarded as a result of repetitive passes of the drug around the blood circulation, for example, [10].

 The identification of the MRT structure revealed the presence of five structural components of MRT, which were denoted by *F*_*cp*_, *F*_*p*_, *F*_*h*_, *F*_*o*_, *F*_*r*_. The structural components relate directly to the drug transport to the blood circulation through the following subsystems: the cardiopulmonary subsystem *H*_*cp*_ [18], the corresponding structural component is *F*_*cp*_, the portal-venous subsystem *H*_*p*_ [19], the corresponding structural component is *F*_*p*_, the hepatic-portal subsystem *H*_*h*_ [20], the corresponding structural component is *F*_*h*_, the subsystem *H*_*o*_ describing drug disposition in noneliminating tissues [21], the corresponding structural component is *F*_*o*_, the subsystem *H*_*r*_, if the drug is subject to the enterohepatic cycling (EHC), for example, [22, 23], the corresponding structural component is *F*_*r*_.

 If the drug is not subject to the EHC, the structural component *F*_*cp*_ can be described as

(2) $$F_{cp}=\frac{Q_{cp}}{Cl_{h}}{\text{MT}}_{cp},$$

where *Q*_*cp*_ is the blood flow in the subsystem *H*_*cp*_, MT_*cp*_ is the mean time of the drug transport through the subsystem *H*_*cp*_, and *Cl*_*h*_ is the hepatic clearance. The structural components *F*_*p*_, *F*_*h*_, and *F*_*o*_ can be described as

(3) $$\begin{array}{c} F_{p}=\frac{Q_{h}-Cl_{h}}{Cl_{h}}\frac{Q_{p}}{Q_{h}}{\text{MT}}_{p},\\ F_{h}=\frac{Q_{h}-Cl_{h}}{Cl_{h}}{\text{MT}}_{h},\\ F_{o}=\frac{Q_{o}}{Cl_{h}}{\text{MT}}_{o}.\; \end{array}$$

In equations above, *Q*_*p*_ is the blood flow in the portal vein, *Q*_*o*_ is the blood flow in noneliminating tissues, MT_*p*_ is the mean time of the drug transport through the subsystem *H*_*p*_, MT_*h*_ is the mean time of the drug transport through the subsystem *H*_*h*_, and MT_*o*_ is the mean time of the drug transport through the subsystem *H*_*o*_, where

(4) $$\begin{array}{c} {\text{MT}}_{o}=\frac{\sum_{i=1}^{q}Q_{i}\cdot {\text{MT}}_{i}}{Q_{o}},\\ Q_{o}=\overset{q}{\underset{i=1}{\sum }}Q_{i}. \end{array}$$

MT_*i*_ is the mean time of the drug transport through a noneliminating tissue; the *i* subscript specifies the tissue [24, 25]. If the drug is subject to the EHC, the structural component *F*_*r*_ can be described as

(5) $$F_{r}=f_{r}\frac{Q_{h}-Cl_{h}}{Q_{h}-f_{r}Cl_{h}}\left({\text{MT}}_{p}+{\text{MT}}_{h}+{\text{MT}}_{r}\right),$$

where MT_*r*_ is the mean time of the drug transport through the subsystem *H*_*r*_, and the coefficient *f*_*r*_, 0 ≤ *f*_*r*_ < 1, determines the fraction of the drug that is the subject to the EHC.

 The resulting equation (6) describes the MRT structure identified

(6) $$\text{MRT}=F_{cp}+F_{p}+F_{h}+F_{o}+F_{r}.$$

From the text above it evident that the right-hand side of the resulting equation (6) is the sum of (2)–(5). Equation (6) looks mathematically elegant and very simple. Nevertheless, this equation provides a mathematical description of the physiologically based structure of the mean residence time of the drug administered as the intravenous bolus to the subject (here to the hypothetical subject, as specified above).

 The transfer function *H*_*iv*_(*s*) used in the general equation (1) is the mathematical relationship between the output and input of the system *H*_*iv*_. Generally, transfer functions are characteristic functions of linear dynamic systems, providing complete descriptions of linear dynamic systems in the Laplace domain, for example, [3–5].

 It is well known that, after an intravenous administration, a total amount of a drug is fully and immediately available to the blood circulation for transports to all areas of the body, a drug is not destroyed by digestive enzymes, an intravenous administration offers an advantage over other routes of administration in its accuracy. The purpose of recalling these well-known facts is to explain why the assumption of the intravenous drug administration was used in the theoretical example in this study.

 The circulatory model developed possesses properties of simplicity and accuracy to describe the disposition of the drug administered as the intravenous bolus to the subject. The properties of the model make the model very flexible because the model is highly capable of accurately describing drug disposition in both situations, that is, when the drug is subject to the EHC and also when the drug is not subject to the EHC. This indicated that the model is very suitable for identifying the MRT structure. The model is very general and appears applicable to several drugs. Using the model, the equations (2)–(6) were determined. Equations (2)–(6) can contribute to understanding mechanisms that control MRT, they can be used to refine already established knowledge of MRT, and can help to gain further insights into physiological background of MRT. The equations (2)–(6) are easy to handle and may be sufficient in pharmacokinetic studies. Primarily this is because these equations are based on the commonly available physiological and pharmacokinetic knowledge.

 The MRT structure identified in this study has not been experimentally validated up to now. Its validity can be verified by further investigations, mainly experimental investigations; consequently a full pharmacokinetic exploitation of the MRT structure identified lies far in the future.

 For the sake of conciseness, mathematical details were restricted to a bare minimum. Differences between traditional pharmacokinetic approaches to MRT and the approach presented in this study were left unexplained. Instead of a comparison of the approaches, this study gives rise to a reasonable expectation that the MRT structure identified may be useful for basic research in pharmacokinetics. This is because the MRT structure identified may reveal features of MRT not apparent from MRT values routinely determined by traditional methods which integrate influences of several processes in the body on MRT into single numerical quantities, not providing any information about physiological backgrounds of MRT.

## 4. Conclusion

 This study presented a new view on “old” principles associated with MRT. It attempted to contribute to the current understanding of MRT, without an intention to criticize traditional approaches to MRT. According to the best of the author's knowledge, and after a Medline search, it can be stated that a physiologically based structure of MRT has not been described in the literature as yet.
